# Correction: Zanin et al. Analysis of Radiation Toxicity in Mammalian Cells Stably Transduced with Mitochondrial *Stat3*. *Int. J. Mol. Sci.* 2023, *24*, 8232

**DOI:** 10.3390/ijms25073672

**Published:** 2024-03-26

**Authors:** Alisa Zanin, Giacomo Meneghetti, Luca Menilli, Annachiara Tesoriere, Francesco Argenton, Maddalena Mognato

**Affiliations:** Department of Biology, University of Padova, Via U. Bassi 58/B, 35131 Padova, Italy

In the original publication [1], there was a mistake in Figure 2b and its legend as published. The representative images of bright-field microscopy of the cell lines MLS_Stat3_NES and MLS_Stat3_NES S727A shared a common area. The 72 h time point was missing in the legend. The corrected Figure 2b and legend appear below. The authors state that the scientific conclusions are unaffected. This correction was approved by the Academic Editor. The original publication has also been updated.

## Figures and Tables

**Figure 2:**
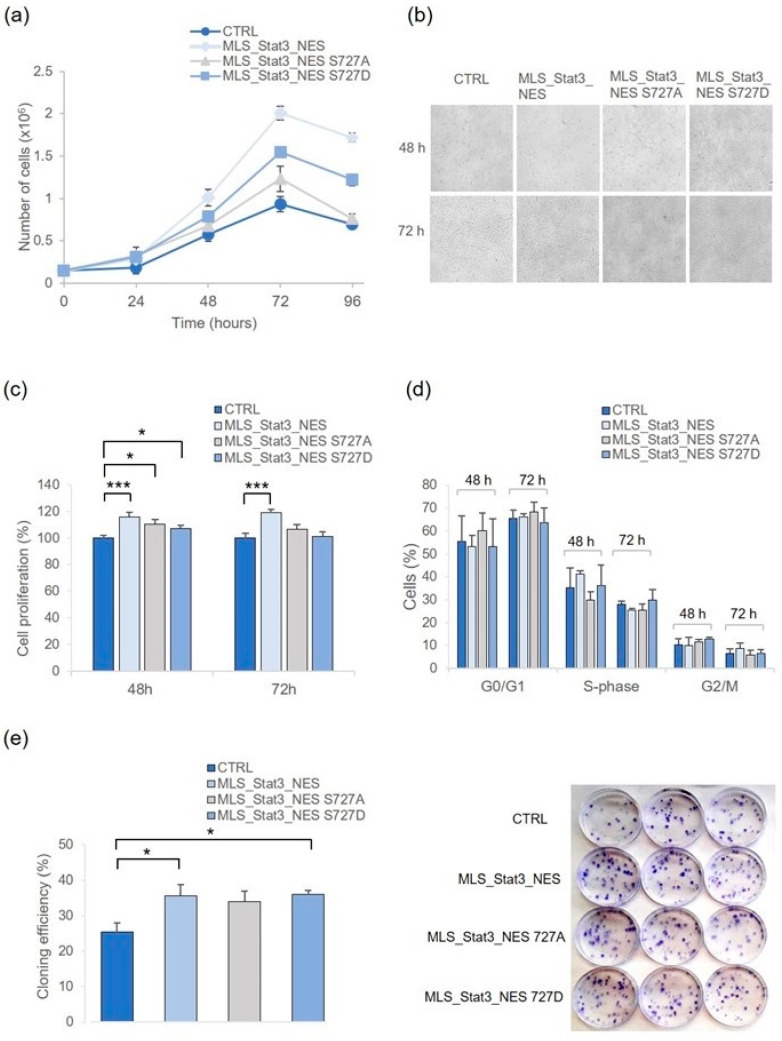
Cell proliferation is enhanced in mitoStat3-transduced NIH-3T3 cells. (**a**) Cell growth curve determined by Trypan blue dye counting at days 1, 2, 3, and 4 after seeding. Data refer to the means ± S.D. of independent experiments (3 ≤ *n* ≤ 6). (**b**) Bright-field microscopy at 48 h and 72 h after seeding; mitoStat3-transduced cells were maintained under selective medium containing G-418 (450 µg/mL). (**c**) Cell proliferation determined at 48 h and 72 h after seeding by MTS. Data are means ± S.E. from independent experiments (3 ≤ *n* ≤ 5; * *p* < 0.05, *** *p* < 0.001, Student’s *t*-test). (**d**) Cell cycle analysis by flow cytometry at the same time points; data were collected from 25.000 cells/sample using a BD LSRFortessa X-20 flow cytometer, and the mean ± S.D. from three independent experiments is indicated. (**e**) Clonogenic assay; data refer to means ± S.E. of independent experiments (3 ≤ *n* ≤ 5), each carried out in quadruplicate (* *p* < 0.05, Student’s *t*-test). Representative images of clones derived from mitoStat3-transduced and non-transduced cells are shown.
