# Supplementary material for: Analysis of Radiation Toxicity in Mammalian Cells Stably Transduced with Mitochondrial Stat3
Source: Int J Mol Sci. 2023 May 4;24(9):8232. doi: 10.3390/ijms24098232 (PMC10179518; doi:10.3390/ijms24098232)
Supplement: Supplementary file 1 [file ijms-24-08232-s001.zip › Figure S1.pdf]

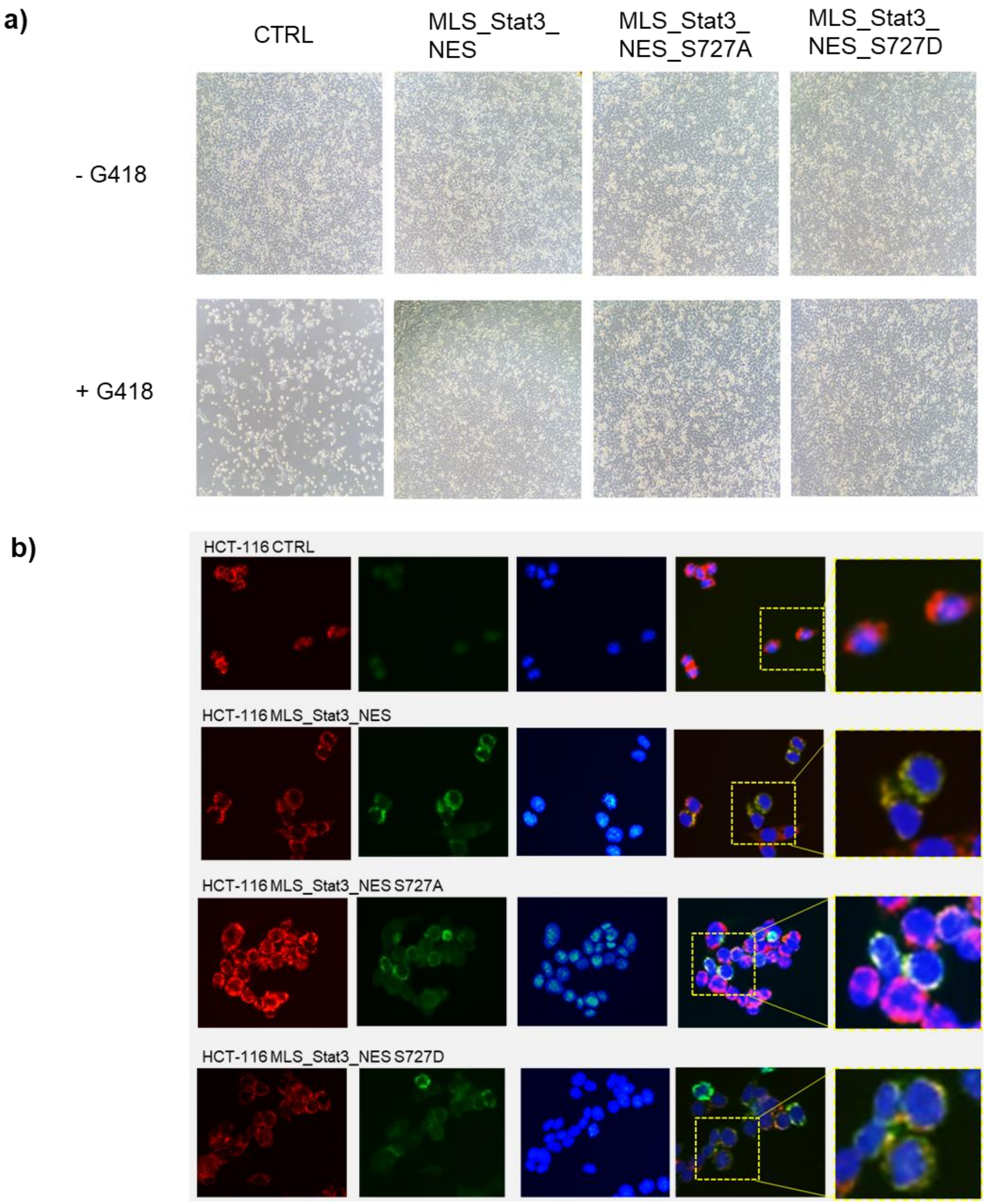

Figure S1. Human HCT-116 cells transduced with mito*Stat3*. **a**) Bright field microscopy at 48 h after seeding; cells transduced with wild-type or mutated at S727 were maintained under selective medium containing G-418 (450µg/mL). **b**) Immunofluorescence analysis of STAT3 cellular localization in cells co-immunostained with anti-STAT3 (green) and anti-ATAD3 (red) as mitochondrial marker. Cell nuclei are counterstained with DAPI (blue). Images were captured under a fluorescent microscope Leica DM6B (40X). Enlarged views of representative merged images are shown.
