# Supplementary material for: Analysis of Radiation Toxicity in Mammalian Cells Stably Transduced with Mitochondrial Stat3
Source: Int J Mol Sci. 2023 May 4;24(9):8232. doi: 10.3390/ijms24098232 (PMC10179518; doi:10.3390/ijms24098232)
Supplement: Supplementary file 1 [file ijms-24-08232-s001.zip › Figure S2.pdf]

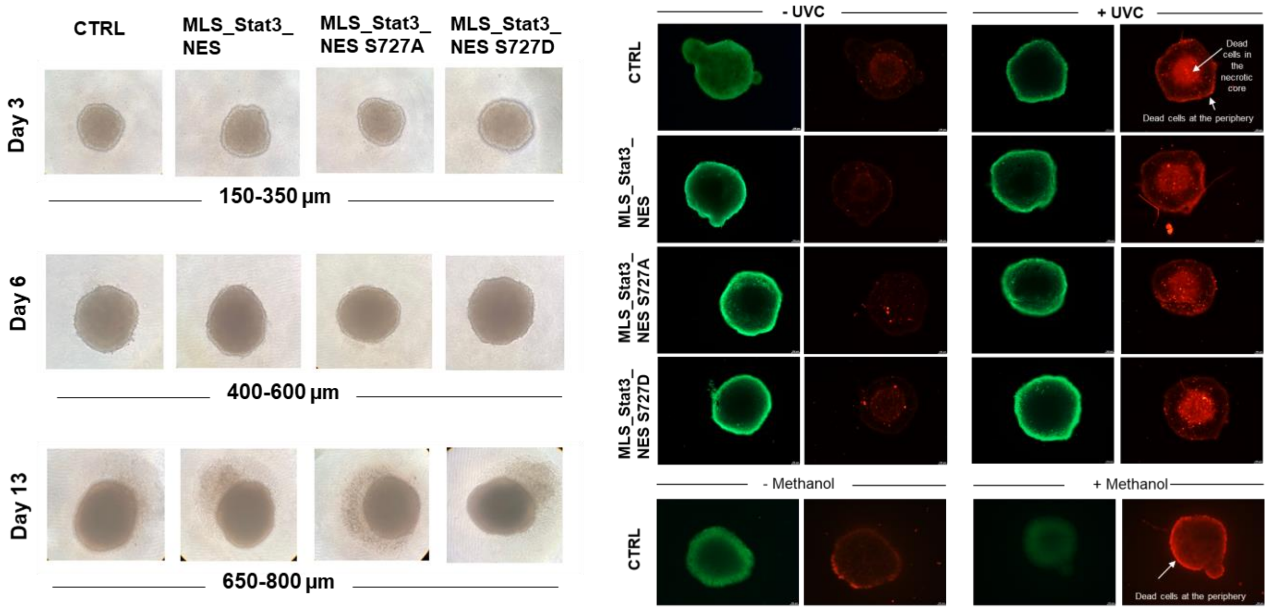

(a) **(b)**

Figure S2. UVC radiation toxicity in HCT-116 cells cultured in 3D. Cells were seeded in pre-coated plates with unique ultra-hydrophilic polymer that enables spontaneous 3D cells formation of uniform size and shape in a scaffold-free modality. After 3 days, we appreciated the self-formation of the spheroids and we observed a spheroids-size increase in time, reaching >200µm at 6-8 days after seeding and the tendency to disassemble with dispersed cells at the periphery at 13 days after seeding. a) Representative images of 3D spheroids of HCT-116 cells at 3, 6 and 13 days after seeding (MO, objective 10X). b) Live-dead assay showing calcein AM/PI staining of live cells (green) and dead cells (red) in 6-days-old spheroids non-irradiated and irradiated with UVC (10 J/m^2) and analysed 24 h after irradiation. Spheroids treated with 70% methanol for 30 minutes showed a bright red fluorescence at the periphery indicating the dead cells. Scalebar represents 100µm.
