# Supplementary material for: Analysis of Radiation Toxicity in Mammalian Cells Stably Transduced with Mitochondrial Stat3
Source: Int J Mol Sci. 2023 May 4;24(9):8232. doi: 10.3390/ijms24098232 (PMC10179518; doi:10.3390/ijms24098232)
Supplement: Supplementary file 1 [file ijms-24-08232-s001.zip › Figure S3.pdf]

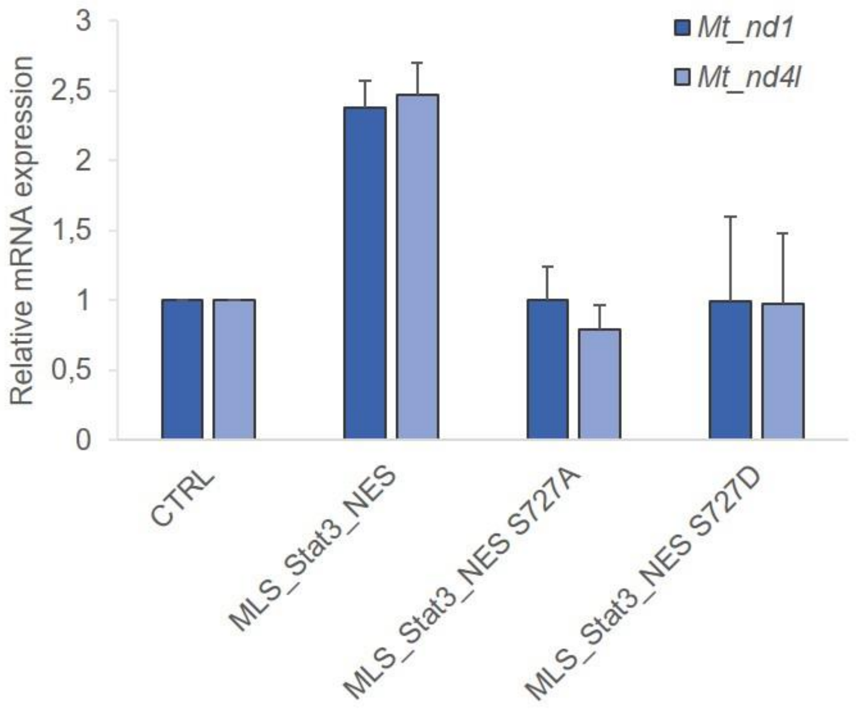

Figure S3. Gene expression analysis of mitochondrial genes *Mt_nd1* and *Mt_nd4l* in mito*Stat3*-transduced NIH-3T3 cells. The relative expression of mRNAs was analysed by qRT-PCR in mito*Stat3*-transduced and non-transduced cells. Bars represent the means ± S.D. of three independent experiments, each performed in triplicate, expressed as fold-change of mito*Stat3*-transduced *vs.* non-transduced cells.
