# Supplementary material for: Analysis of Radiation Toxicity in Mammalian Cells Stably Transduced with Mitochondrial Stat3
Source: Int J Mol Sci. 2023 May 4;24(9):8232. doi: 10.3390/ijms24098232 (PMC10179518; doi:10.3390/ijms24098232)
Supplement: Supplementary file 1 [file ijms-24-08232-s001.zip › Figure S4.pdf]

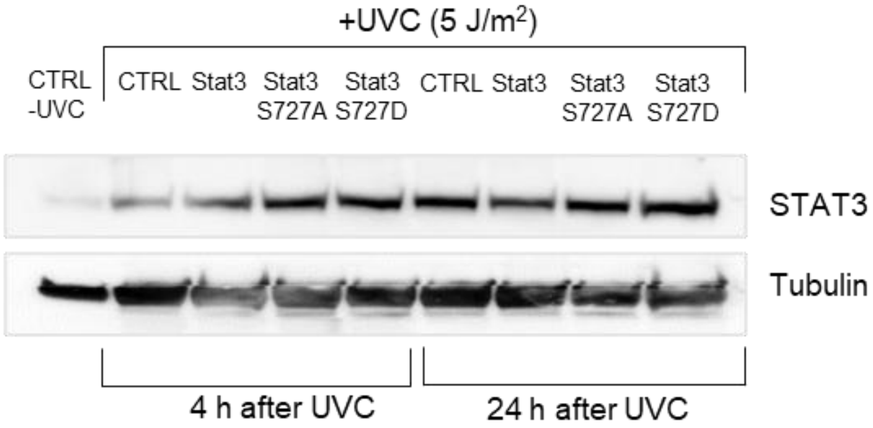

Figure S4. Expression of STAT3 protein in NIH-3T3 cells after irradiation with UVC. Western blot was performed on whole cell lysates of mito*Stat3*-transduced and non-transduced cells at 4 and 24 h after irradiation with UVC (5 J/m^2). Tubulin was used as loading control.
