# Supplementary material for: Analysis of Radiation Toxicity in Mammalian Cells Stably Transduced with Mitochondrial Stat3
Source: Int J Mol Sci. 2023 May 4;24(9):8232. doi: 10.3390/ijms24098232 (PMC10179518; doi:10.3390/ijms24098232)
Supplement: Supplementary file 1 [file ijms-24-08232-s001.zip › Supplementary Table S1.pdf]

Supplementary Table S1. Sequence of primers used for gene expression analysis by qRT-PCR.

**Gene**	**Primer**	**Sequence**
m*Gadd45a*	FW	CTGTGTGCTGGTGACGAAC
	RV	TCCATGTAGCGACTTTCCCG
m*Ddb2*	FW	CAACCAGGCTGCAGGATTTT
	RV	TGGCAGAAACATCAAGGCTG
m*Tp53*	FW	CCAAGTCTGTTATGTGCACGT
	RV	CTGACCCACAACTGCACAG
m*P21*	FW	CATGGGTTCTGACGGACATC
	RV	GTCTCGGTGACAAAGTCGAAGTT
m*Stat3*	FW	TGTTGGAGCAGCATCTTCAG
	RV	GAGGTTCTCCACCACCTTCA
m*Gapdh*	FW	CATCACTGCCACCCAGAAGACTG
	RV	ATGCCAGTGAGCTTCCCGTTCAG
